# Study of Application of an Active Ultrasound by Use of Zn-Al-Mg-Ti-Based Solder on Selected Substrates

**DOI:** 10.3390/ma18051094

**Published:** 2025-02-28

**Authors:** Roman Koleňák, Tomáš Meluš, Jaromír Drapala, Peter Gogola, Matej Pašák

**Affiliations:** 1 Faculty of Materials Science and Technology in Trnava, Slovak University of Technology in Bratislava, Jána Bottu No. 2781/25, 917 24 Trnava, Slovakia; peter.gogola@stuba.sk (P.G.); matej.pasak@stuba.sk (M.P.); 2 Department of Non-Ferrous Metals, Refining and Recycling, FMT-Faculty of Materials Science and Technology, Technical University of Ostrava, 17. Listopadu 15, 708 33 Ostrava, Czech Republic; jar.drapala@seznam.cz

**Keywords:** soldering, Al_2_O_3_ ceramic, Cu substrate, Zn solder, ultrasonic soldering

## Abstract

This study investigates the potential application of Zn5Al1.5Mg1.5Ti active solder in ultrasonic soldering of Al_2_O_3_ ceramics and Cu substrates. The research explores the microstructural characteristics, phase composition, and mechanical properties of the solder and the resulting joints. Particular attention is given to the formation mechanisms of the solder–substrate bond and the role of ultrasound activation in enhancing wettability and bond strength. The study aimed to provide a deeper understanding of active soldering processes and their suitability for high-temperature applications. The findings contribute to advancing lead-free soldering technologies for electronic and structural applications.

## 1. Introduction

Soldering alloys are extremely important in the electronic industry, where they are used for soldering of electronic components and printed circuit boards (PCBs). Traditional high-temperature solders, such as Pb5Sn and Pb10Sn, are widely used in electronic packages and the automotive, aviation, space and power industries [1,2]. Sn-Pb-based solders are often used in electronic equipment due to their reliability and durability. However, lead is toxic and harmful to health, so manufacturers are increasingly required to use alternative soldering alloys [3,4].

Research into soldering materials in this field has advanced considerably, leading to the development of many other soldering alloys that can replace traditional lead-based alloys. Several possible alloy systems meet these criteria, including those based on gold, bismuth or silver; however, their high price significantly limits their application. Zinc-based alloys, on the other hand, seem to be a suitable alternative, offering high strength, good thermal and electrical conductivity and high reliability, among other advantages [5,6,7].

As a substitute for high-temperature solders, each of these alloys presents certain pros and cons. Based on these considerations, zinc-based alloys seem to be the most suitable. These alloys are not only affordably priced but also provide excellent strength, efficient thermal and electric conductivity and high-level reliability. Existing sources highlight Al-Zn-based solders in particular, which are characterized by an ideal melting point range, corrosion resistance and impressive mechanical properties [8,9,10].

In conclusion, these alloys represent promising options for applications in the pro-duction of power semiconductors, optical devices and diverse electronic applications [11,12,13]. Zinc-based alloys have been proposed as promising lead-free solders due to their low cost, low coefficient of thermal expansivity and good electrical and thermal conductivity [14,15,16]. The addition of Mg, Cu and Ga can significantly lower the melting point of Zn-Al-based alloys [16,17,18]. For example, the Zn4Al3Mg alloy (wt.%) has an approximate eutectic temperature of 343 °C, with the melting point further reduced by adding a fourth element [18,19,20].

Cui et al. [21] studied the soldering of sapphire using Zn4Al solder with ultrasonic activation. The bond between sapphire and its interlayer was formed due to the ultrasound application. To reduce the coefficient of thermal expansion (CTE) of the soldering metal, an interlayer of SiCp/A356 composites was applied, creating a sandwich structure of Zn4Al/(SiCp/A356)/Zn4Al. During coating and soldering with ultrasonic assistance, the composite matrix dissolved in the molten Zn4Al alloy. A maximum shear strength of approximately 155 MPa was achieved. Fracture morphology suggests a strong bond between the Zn4Al alloy and sapphire, with a mixed fracture mechanism shown in the shear test.

Haque et al. [22] selected a base metal consisting of zinc and aluminum. They mixed zinc, aluminum, magnesium and gallium to create a Zn4Al3Mg3Ga alloy. The substrates used were copper and silicon with a Ti/Ni/Ag coating. Soldering was performed in the temperature range of 370 to 400 °C using automated equipment for chip mounting. The ZnAlMgGa/Cu joints achieved tensile strengths ranging from 21.8 to 29.4 MPa at soldering temperatures of 370 to 400 °C.

The study by Chen et al. [23] investigated the direct soldering of SiC ceramics with ultrasonic assistance. The SiC ceramic substrates were soldered in air using a Zn8.5Al1Mg solder at a temperature of 420 °C. The tensile strength of the joints increased with extended ultrasound application time, with the highest tensile strength (148.1 MPa) achieved after 8 s of ultrasonic assistance. A new amorphous layer, 2 to 6 nm thick, was formed at the boundary between the solder and the substrate. Atoms from the eroded SiO2 layer on the SiC substrate surface rapidly diffused into the solder due to the effects of ultrasound. The strong bond between the SiC substrate and the Zn-Al-Mg solder is explained by the transfer of SiO_2_ into the Zn8.5Al1Mg solder, driven by cavitation erosion caused by ultrasound.

The authors’ goal [24] was to investigate the Zn-In-Mg alloy and its direct soldering with silicon and copper. This solder has a wide melting range, dependent on the indium content. Its microstructure consists of a pure Zn matrix, with MgZn_2_ and (In)β-In phases at the grain boundaries. The tensile strength ranges from 46 to 124 MPa, depending on indium content. The bond with silicon forms through reactions of In and Mg with the substrate. Diffraction studies revealed an In13Mg7 phase. The copper bond is not influenced by indium or magnesium, forming through the interaction between zinc and copper. Two phases, CuZn_4_ and Cu_5_Zn_8_, were observed. The shear strength of Cu/Zn-In-Mg/Cu joints ranges from 56 to 62 MPa, and the shear strength of Si/Zn-In-Mg/Cu joints is between 34 and 42 MPa.

The authors’ study [25] focused on direct brazing of a metal–ceramic composite (MMC) with copper substrate without flux. The brazing was carried out with Zn-type Zn10In1Mg solder. The solder joints were fabricated using power ultraction. The solder used consisted of a zinc matrix, while the solid solution (In) and the MgZn_2_ phase were separated at the grain boundaries. The soldered MMC joint was formed by dissolving the hli–nickel matrix in the zinc solder. This resulted in a new composite composed of a matrix pozzolanic predominantly from solid solution (Al). In addition, a solid solution (In) and a Cu_3.2_Zn_0.7_Al_4.2_ phase were also present. The bond with the copper substrate was formed by the interaction of Zn and Al from the solder to form two transition phases, namely Cu_3.2_Zn_0.7_Al_4.2_ and the unstable Al(Cu,Zn)_2_ phase. The average shear strength of the MMC/Cu combi-joints was 16.5 MPa.

The research in this work focused on the characterization of Zn5Al1.5Mg1.5Ti solder intended for high-temperature applications. The microstructure, phase composition, temperature of phase transformations and tensile strength were studied. This work explores the use of Zn5Al1.5Mg1.5Ti solder for ultrasonic soldering of Al_2_O_3_ ceramics and Cu substrate. The study aimed to assess the characteristics of the soldered joints through the analysis of the solder–substrate boundary. The strength of the fabricated joints was evaluated by the shear testing. This research provides a new understanding into ultrasonic soldering of ceramic/copper combinations using a Zn5Al1.5Mg1.5Ti solder.

## 2. Experimental

The soldering alloy was prepared as follows: a thin Ti foil was dissolved in Zn in an induction oven. In a separate experiment, ZnTi2 pre-alloy and other alloys were melted in Zn using an induction oven, followed by casting into a graphite crucible without a shielding atmosphere.

The chemical composition given in Table 1 was prepared experimentally. Chemical analysis was performed using inductively coupled plasma atomic emission spectroscopy (ICP-AES), also referred to as inductively coupled plasma optical emission spectroscopy (ICP-OES), an analytic technique for detecting chemical elements using a SPECTRO VI-SION EOP instrument (SPECTRO Analytical Instruments, Kleve, North Rhine-Westphalia, Germany). This method is a type of emission spectrometry that utilizes inductively coupled plasma to produce excited atoms and ions, which emit electromagnetic radiation at wavelengths characteristic of specific elements.

The melting point of the soldering alloy was measured using differential thermal analysis (DTA). This analysis was performed on a DTA SETARAM Setsys 18TM instru-ment (KEP Technologies Inc., Austin, TX, USA). The DTA analyses were performed under the following conditions: Ar atmosphere (5N purity), Ar flow rate of 50 mL/min and temperature increment of 5 °C/min. These temperatures were recorded and analyzed using the SETSOFT software on a PC (version 4.2, SETARAM Instrumentation, Caluire, Rhône, France). From the resulting DTA curves, the phase transformation temperatures within the liquidus–solidus range were determined.

XRD measurements were carried out on metal sawdust derived from the cast and annealed soldering alloy samples using a PANalytical Empyrean X-ray diffractometer (XRD, Malvern PANalytical Ltd., Malvern, UK). Sawdust, rather than bulk castings, was employed to avoid the influence of casting texture on the recorded XRD patterns. The measurements were conducted in Bragg–Brentano geometry, with an angle range of θ–2θ between 20° and 145°. The XRD system was powered by a co-anodic lamp, set to 40 kV and 40 mA. The incident beam was adjusted with a 0.04 radian Soller slit, 1/4° divergence slit, 1/2° anti-scatter slit, a Fe beta filter and a PIXcel3D position-sensitive detector, operating in a 1D scanning mode. Phase identification was performed using PANalytical Xpert High Score software (version 3.0.5) with the ICSD FIZ Karlsruhe database.

**Figure 1:**
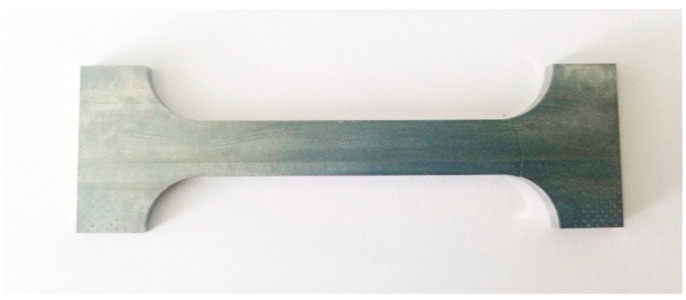
Shape and dimensions of the test piece for tensile strength measurement.

The mechanical tests were conducted to determine the tensile strength of the active soldering alloy Zn5Al1.5Mg1.5Ti. In accordance with standard experimental practice, a minimum of three test specimens per geometry were used to ensure the repeatability and reliability of the obtained data. The specimens were of standardized shape and dimensions, as shown in Figure 1, with a thickness of 4 mm.

The tensile tests were performed using a LabTest 5.250SP1-VM universal testing machine, with a controlled loading rate of 1 mm/min. During the tests, force and displacement signals were continuously recorded in real time to capture the mechanical response of the solder. The obtained force–displacement curves were analyzed, and stress–strain relationships were derived using these standard formulas:

$$\sigma =\frac{F}{A}$$

$$\varepsilon =\frac{\Delta L}{L_{0}}$$

where σ is the stress, *F* is the applied force, *A* is the initial cross-sectional area of the specimen, ε is the strain, Δ*L* is the elongation and *L*_0_ is the original length of the specimen.

The accuracy of the force measurement system was ±0.5% of the full-scale load, and the displacement accuracy was ±0.02 mm. The collected data were further processed to generate stress–strain curves, which provide a more detailed understanding of the mechanical behavior of the tested solder joints.

The microstructure was assessed using a JEOL JSM 7600F scanning electron micro-scope (SEM/EDX, JEOL Ltd., Tokyo, Japan) with a Schottky field emission electron source operating at 20 kV and 90 µA. The specimens were positioned at a working distance of 15 mm and analyzed using a backscatter electron detector. Elemental analysis was per-formed using an Oxford Instruments X-Max silicon drift detector and an energy dispersive X-ray spectrometer (EDS, Oxford Instruments plc, Abington, UK).

Soldering was performed on substrates with the following dimensions:

- Al_2_O_3_ ceramics in disk form with diameter Φ 15 mm × 3 mm,
- Cu material in disk form with diameter Φ 15 mm × 3 mm,
- Cu material in cube form with dimensions 10 × 10 × 3 mm.

**Figure 2:**
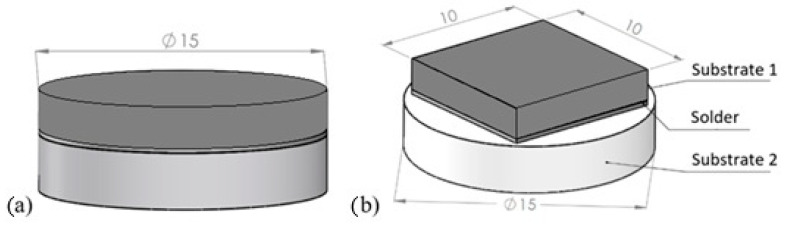
Soldered joint assembly (**a**) for analysis of solder–substrate boundaries; (**b**) for shear strength measurement.

Figure 2a shows a schematic of the soldered joint for analyzing the solder–substrate boundaries, and the schematic for measuring shear strength is shown in Figure 2b.

Solder activation was performed using an ultrasonic device, the Hanuz UT2-ultrasonic transducer, which utilizes an oscillating piezoelectric system with a titanium sonotrode that is 3 mm in diameter. Ultrasound parameters are given in Table 2. Heating of the solder was performed on a CERAN 500 hot plate.

The soldering procedure consisted of degreasing Al_2_O_3_ and Cu substrates, which were laid on a hot plate. Heating of the hot plate was set according to the melting point of the soldering alloy used. Joints were fabricated using an active solder of type Zn5Al1.5Mg1.5Ti, and a small amount of solder was subsequently deposited on the substrate surface.

**Figure 3:**
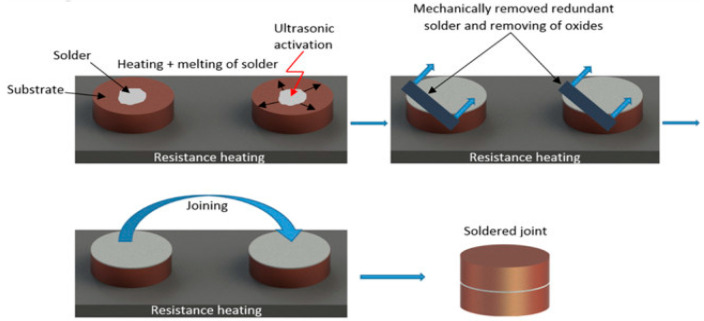
Schematic representation of ultrasonic soldering.

The next step consisted of immersing the sonotrode of the soldering equipment into the molten solder for 5 s. Due to the effect of ultrasonic vibrations, the solder was activated, and the oxides in the soldering alloy were gradually disrupted and subsequently washed off of the solder surface. The prepared surfaces, along with the molten solder, were then brought together to form the bond. This joint assembly was then left to cool freely at room temperature. Figure 3 shows the schematic procedure of ultrasonic soldering.

Metallographic preparation consisted of the processes of grinding, polishing and etching the embedded specimens. The specimens were clamped in a jig for grinding, which was performed using diamond emery papers with grit sizes of 600, 1200 and 2400. During the grinding process, water was supplied to the emery paper to wash away debris. Each grinding stage with each emery paper lasted 3 min.

After grinding the specimens, the polishing process was carried out using polishing discs with diamond emulsions with particle sizes of 9 μm, 6 μm, 3 μm and 1 μm, with each emulsion applied for 10 min. After grinding and polishing of the specimens, the process of coating with carbon followed to ensure a conductive surface suitable for SEM analysis.

The special clamping fixture was used to hold the specimen, ensuring that its interface was parallel to the direction of the compressive force. The opposing parts of the fixture were inserted into a guiding tube to prevent any misalignment. Subsequently, the fixture was secured in the jaws of the testing device, and the load was applied at a closing speed of 1 mm/min until the specimen failed. After each test, the specimen was removed and replaced with a new one.

**Figure 4:**
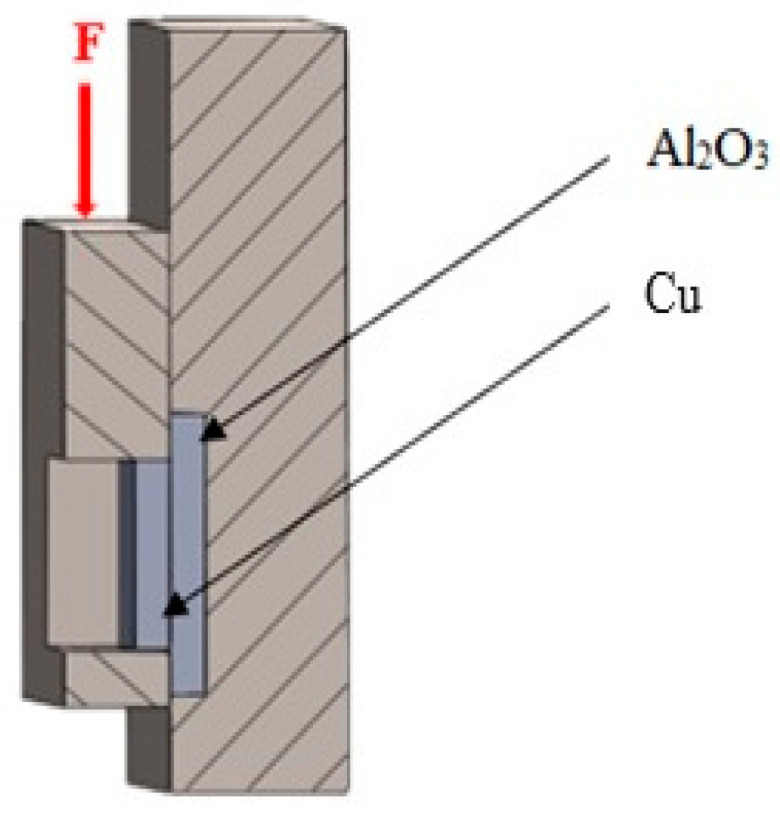
Schematic representation of a jig for shear strength measurement.

The shear strength was determined using a LabTest 5.250SP1-VM universal testing machine. To change the direction of the loading force acting on the test specimen, a jig with a specific shape was used (Figure 4). This shearing jig ensured uniform shear loading at the solder–substrate interface. Three joints were tested to measure shear strength.

## 3. Experimental Results

### 3.1. DTA Solder Analysis

**Figure 5:**
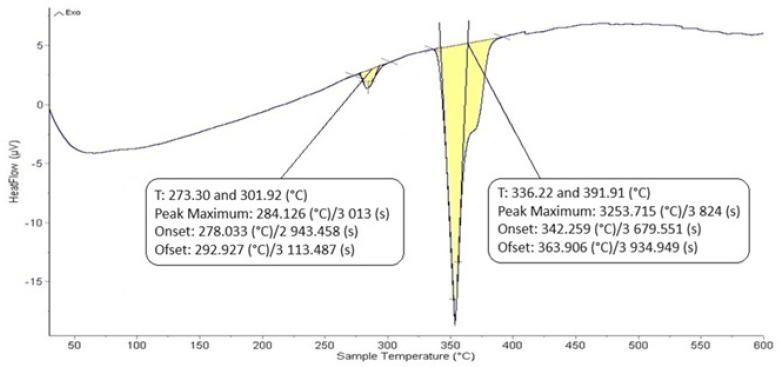
DTA analysis of Zn5Al1.5Mg1.5Ti solder; the heating rate was 5 °C/min.

**Figure 6:**
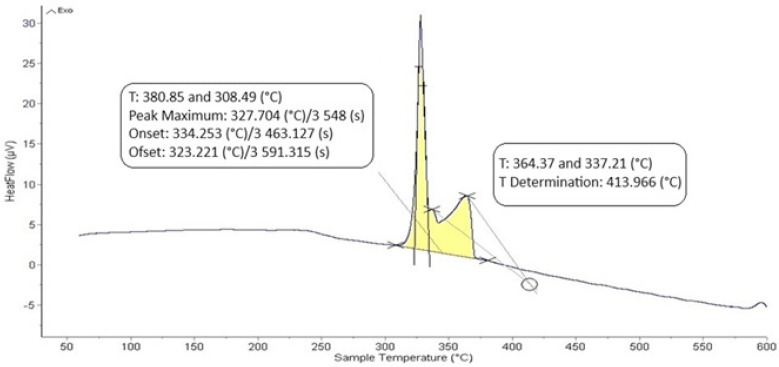
DTA analysis of Zn5Al1.5Mg1.5Ti solder; the cooling rate was 5 °C/min.

The DTA analysis of the Zn5Al1.5Mg1.5Ti solder is shown in Figure 5 and Figure 6 and illustrates the existence of two significant transformations. The ZnAl5Mg1.5Ti1.5 alloy is comprised of four elements, with several phases identified in its structure. Titanium was observed in several modifications, primarily as the intermetallic compound TiAl_3_, which was initially precipitated. Subsequently, a ternary phase, known as phase T, has a constant content of titanium, while the zinc and aluminum content can vary (see Figure 8).

The intermetallic phase MgZn_2_ was another structural component. Additionally, a eutectoid mixture of solid solutions (Al) + (Zn) was identified, while the matrix was formed of the solid solution (Zn). The difference between the eutectic temperature obtained from the binary phase diagram of Al-Zn (361 °C) and the temperature determined by DTA analysis (342 °C) is attributed to the presence of magnesium (approximately 10 at.%) in the ternary eutectics (see Figure 8) and the EDX analysis in Table 3. The ternary eutectic was composed of a mixture of (Zn) + (Al) + (Mg), with relatively high Mg and Al contents. The presence of magnesium lowered the eutectic temperature. Figure 5 and Figure 6 suggest that the DTA analysis confirmed the existence of a reaction at approximately 380 °C, observed in both heating curve measurements.

### 3.2. Microstructure of the Solder Bi11Ag1Mg

**Figure 7:**
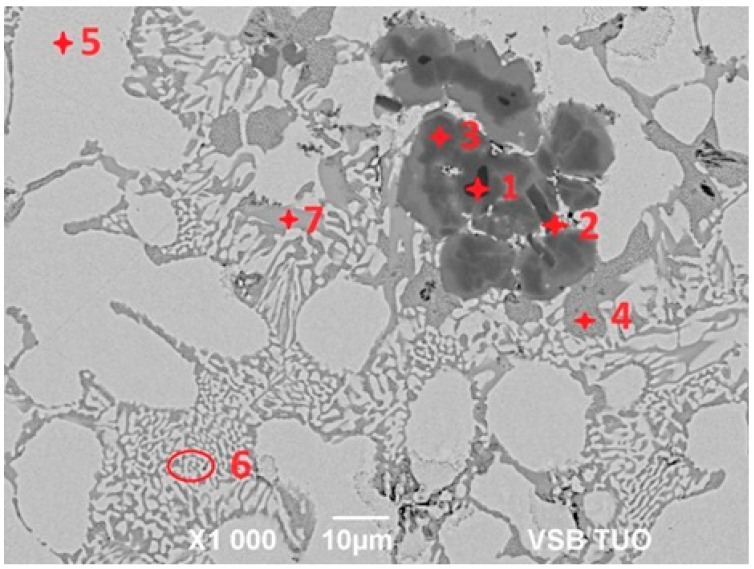
Microstructure of Zn5Al1.5Mg1.5Ti solder.

For determination of the chemical composition and identification of individual phases of Zn5Al1.5Mg1.5Ti solder (see Figure 7), SEM/EDX analysis of spectrum 1 through 7 were performed (see Table 3). Spot 1 represents a dark section, containing 70.8 at.% Al, 24.4 at.% Ti and 4.8 at.% Zn, corresponding to the intermetallic phase TiAl_3_. The dark-grey area at spot 2 contained 26.5 at.% Al, 67.7 at.% Ti and 5.8 at.% Zn, which is associated with the Ti_3_Al phase, exhibiting a considerably wide concentration range of interactions.

**Figure 8:**
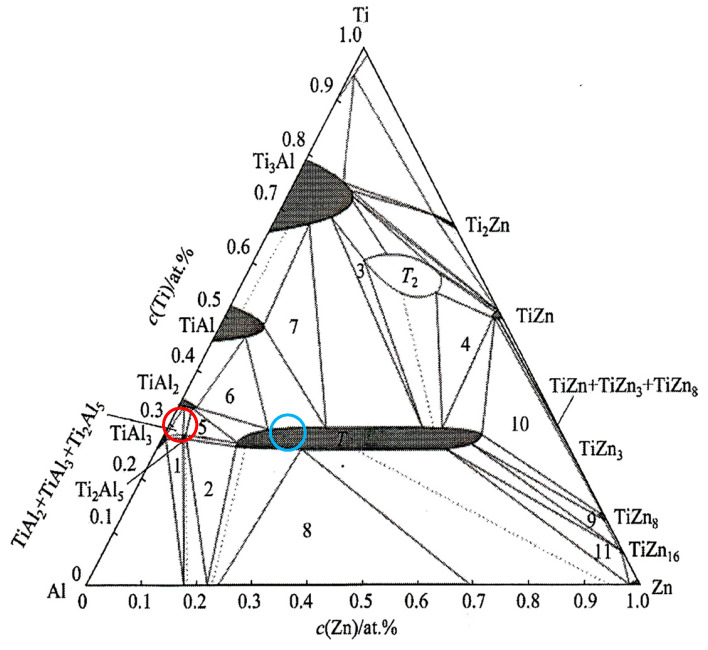
Isothermal section of a ternary diagram of titanium–aluminum–zinc. Red point—spectrum 3, blue point—spectrum 4. Point 1—TiAl_3_ + Ti_2_Al_5_ + α(Al); point 2—Ti_2_Al_5_ + α(Al) + T; point 3—T + T_2_ + Ti_3_Al; point 4—T + T_2_ + TiZn; point 5—Ti_2_A_l5_ + TiAl_2_ + T; point 6—T + TiAl_2_ + TiAl; point 7—Ti_3_Al + TiAl + T; point 8—Liquid + α(Al) + T; point 9—T + TiZn_8_ + TiZn_16_; point 10—T + TiZn + TiZn_8_; point 11—T + TiZn_16_ + Liquid [26,27].

The dark-grey section in spectrum 3 contained 41.0 at.% Al, 24.9 at.% Ti and 35 at.% Zn. In this case, the ternary T phase, identified as TiAl_3_, is relevant (see Figure 8, marked with a red circle).

The brighter grey area in spot 4 contained, on average, 32.6 at.% Mg, 2.6 at.% Al and 64.8 at.% Zn. Based on the phase diagram, this is indicative of the intermetallic phase MgZn_2_, which has a relatively high melting point. In this phase, approximately 2 at.% Al was dissolved.

Spectrum 5 contained, on average, 40.5 at.% Al and 59.5 at.% Zn. This corresponds to the eutectoid mixture of solid solutions (Al) + (Zn) formed by the eutectoid reaction at 277 °C, with a eutectoid concentration of 59 at.% Zn, which aligns well with the overall composition of the spot.

The bright areas in spectrum 6 contained, on average, 96.9 at.% Zn, with the balance being Al, indicating the presence of a solid solution of zinc.

The brighter grey areas in spectrum 7 contained 59.7 at.% Al, 23.8 at.% Ti and 16.5 at.% Zn. Again, this corresponds to the ternary T phase, identified as TiAl_3_ (see Figure 8, marked with a blue circle). The ternary phase (see Figure 8) contains approximately 25 at.% Ti, with the aluminum content varying from 15 at.% Al to 60 at.% Al. Therefore, the T phase is stable over a wide range of Al and Zn concentrations at a constant titanium content of approximately 25 at.%.

**Table 3:** Results from the point SEM/EDX analysis performed on the soldering alloy type Zn5Al1.5Mg1.5Ti.

Spectrum	Mg [at. %]	Al [at. %]	Ti [at. %]	Zn [at. %]	Solder Component
1	-	70.8	24.4	4.8	intermetallic phase TiAl_3_
2	-	26.5	67.7	5.8	Ti_3_Al phase
3	-	40.1	24.9	35.0	T-type ternary phase TiAl_3_
4	32.6	2.6	-	64.8	MgZn_2_ intermetallic phase
5	-	40.5	-	59.5	solid solution (Al) + (Zn)
6	-	3.1	-	96.9	solid solution (Zn)
7	-	59.7	23.8	16.5	T-type ternary phase TiAl_3_

**Figure 9:**
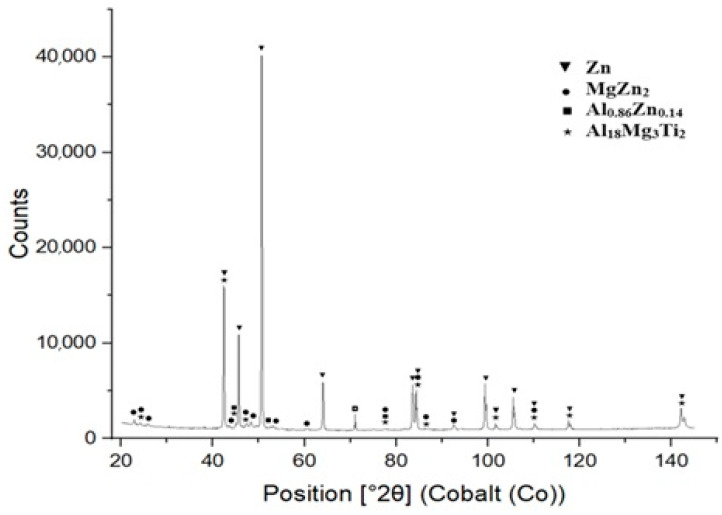
XRD analysis of Zn5Al1.5Mg1.5Ti solder.

XRD measurements were performed using an energy dispersive X-ray spectrometer on specimens prepared from the bulk solder samples. The XRD analysis confirmed the presence of intermetallic phases, namely MgZn_2_, Al_0.86_Zn0.14 and Al1_8_Mg_3_Ti_2_. The results from the diffraction analysis are documented in Figure 9.

### 3.3. Tensile Strength of the Solder Alloy

The mechanical tests focused on measuring the tensile strength of the active soldering alloy type Zn5Al1.5Mg1.5Ti. The dimensions of the test pieces were designed and calculated accordingly. Three specimens were used for tensile strength measurement of the experimental solder, with a loading rate of 1 mm/min for each specimen. For comparison, measurements were also conducted on another soldering alloy, Zn5Al1.5Mg, to evaluate the effect of elemental additions on the final strength of the experimental alloy.

The obtained results for Zn5Al1.5Mg1.5Ti are summarized in Table 4. The minimum tensile strength was 71 MPa, while the maximum tensile strength reached 108 MPa. The average tensile strength calculated from the measured values was 89.5 MPa, and the median tensile strength was 89.5 MPa. The calculated range between the lowest and highest strength was 37 MPa. The standard deviation of the tensile strength measurements was 26.16 MPa, indicating some variation between the tested samples.

**Figure 10:**
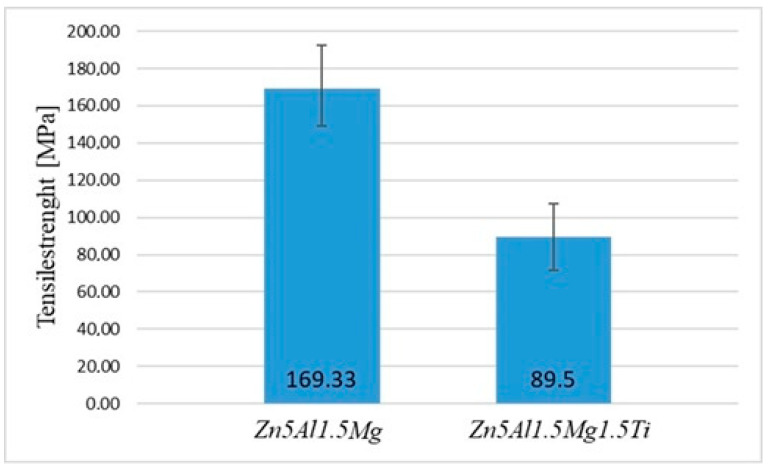
Tensile strength of experimental soldering alloys.

From the results of measurements shown in Figure 10, it is evident that the highest strength values were achieved for the Zn5Al1.5Mg solder without titanium addition, where the highest average tensile strength was 169.33 MPa. The results indicate that the addition of 1.5 at.% Ti to the Zn–Al–Mg soldering alloy significantly reduces the average tensile strength by nearly 45%, decreasing from 169.33 MPa (Zn5Al1.5Mg) to 89.5 MPa (Zn5Al1.5Mg1.5Ti). This substantial decrease suggests that although Ti enhances the wettability and bonding at the interface, it negatively affects the mechanical properties of the alloy. The ductility of the test pieces for the Zn5Al1.5Mg1.5Ti alloy varied from 0.15 to 0.29%.

These findings align with previous studies on Zn-based solder alloys, which also reported a strength reduction with Ti addition. Understanding this trade-off is crucial for optimizing the composition of advanced solder materials used in high-temperature applications, particularly in power electronics, aerospace and automotive industries, where the reliability of solder joints is critical.

**Table 4:** Summary of tensile strength test results for Zn5Al1.5Mg1.5Ti.

Test Type	Solder Type	Number of Samples	Min [MPa]	Max [MPa]	Median [MPa]	Range [MPa]	Standard Deviation [MPa]
Tensile Strength	Zn5Al1.5Mg1.5Ti	3	71	108	89.5	37	26.16

### 3.4. Analysis of Transition Zone Between the Joint of Al_2_O_3_/Zn5Al1.5Mg1.5Ti

**Figure 11:**
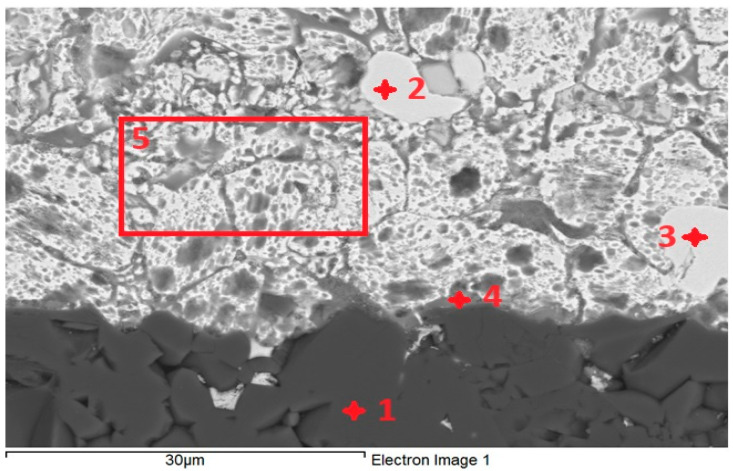
EDX point analysis of Al_2_O_3_/Zn5Al1.5Mg1.5Ti joint.

For the determination of chemical composition and identification of individual phases, SEM/EDX analysis was performed. The transition zone in the Al_2_O_3_/Zn5Al1.5Mg1.5Ti joint (Figure 11) was analyzed in more detail. The regions from 1 to 5, as given in Table 5, were analyzed. Location 1 stoichiometrically represents the Al_2_O_3_ substrate and corresponds well to the following proportion of elements: Al:O = 2:3, or 40:60.

According to the Cu-Zn phase diagram, locations 2 and 3 undoubtedly represent the CuZn4 phase, which is slightly contaminated with aluminum oxide.

The point analysis of location 4 reveals the presence of Al_2_O_3_ from the substrate and solder (20 at.% Zn, 0.3 at.% Ti, variable content of Mg and 2.8 at.% Cu from the substrate).

The analysis of the soldering surface in location 5 indicates that the content of Mg and Al correspond to the nominal composition of the solder.

**Table 5:** Results from the point energy–dispersion analysis of Al_2_O_3_/Zn5Al1.5Mg1.5Ti joint.

Spectrum	O [at.%]	Mg [at.%]	Al [at.%]	Ti [at.%]	Cu [at.%]	Zn [at.%]	Solder Component
1	60.31	0.06	39.55	-	-	0.08	Substrate Al_2_O_3_
2	2.33	0.46	2.26	-	16.78	78.17	CuZn_4_
3	2.47	-	2.53	-	16.88	78.11	CuZn_4_
4	42.84	0.96	31.87	0.38	2.95	21.01	-
5	23.05	3.44	8.68	0.21	2.95	61.68	Surface analysis

**Figure 12:**
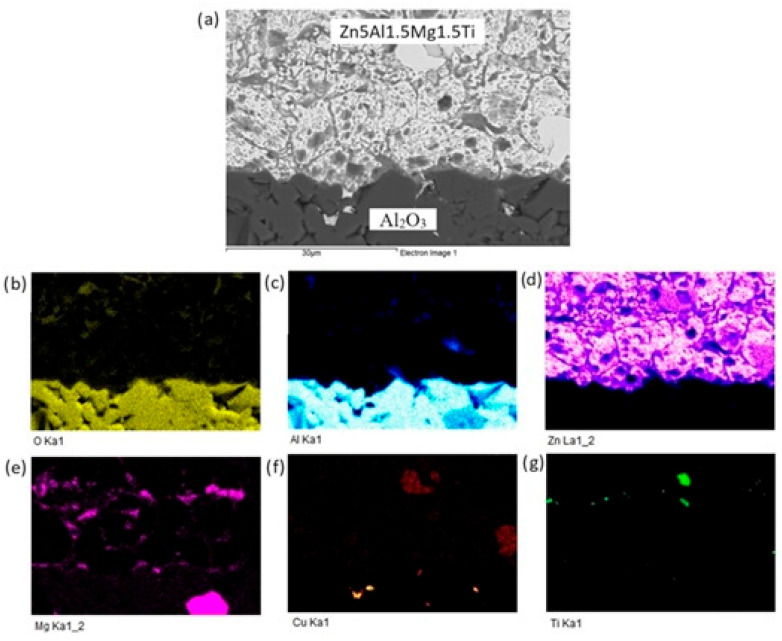
Planar distribution of elements in the boundaries of Al_2_O_3_/Zn5Al1.5Mg1.5Ti: (**a**) joint microstructure; (**b**) Ti; (**c**) O; (**d**) Al; (**e**) Zn; (**f**) Mg and (**g**) Cu.

The planar distribution of elements at the boundary is documented in Figure 12. Viewing this distribution, it is obvious that magnesium significantly contributes to bond formation. The violet color indicates the presence of magnesium at the boundary.

**Figure 13:**
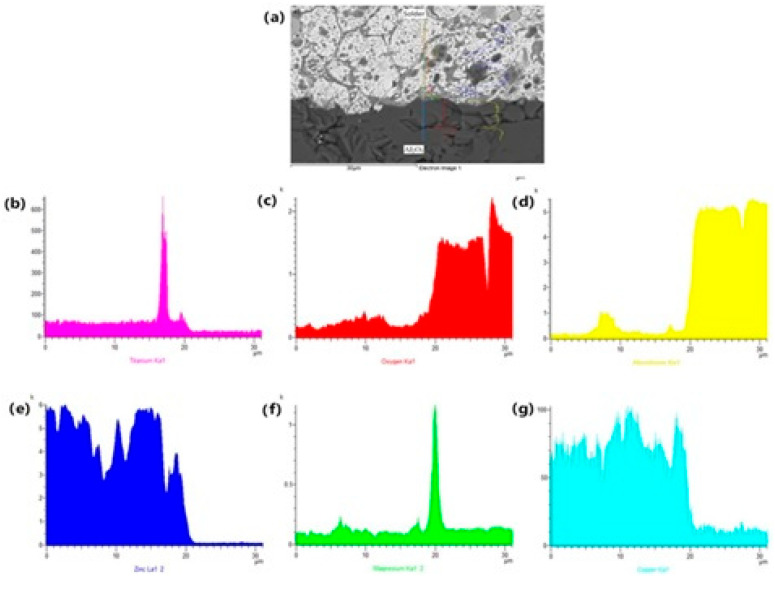
The line EDX analysis of soldered joint of Al_2_O_3_/Zn5Al1.5Mg1.5Ti: (**a**) joint microstructure; (**b**) Ti; (**c**) O; (**d**) Al; (**e**) Zn; (**f**) Mg and (**g**) Cu.

The EDX analysis shown in Figure 13 represents the concentration profile across the boundary of the Al_2_O_3_/solder joint, covering its entire length of 30 μm. Due to the interaction between the solder and the Al_2_O_3_ substrate during soldering, an increase in Mg content occurred in a very narrow layer at the boundary, approximately 1 to 2 µm wide. A significant Mg peak is observable, indicating its segregation at the ceramic/solder boundary.

### 3.5. Analysis of Transition Zone Between the Soldered Joint of Zn5Al1.5Mg1.5T/Cu

**Figure 14:**
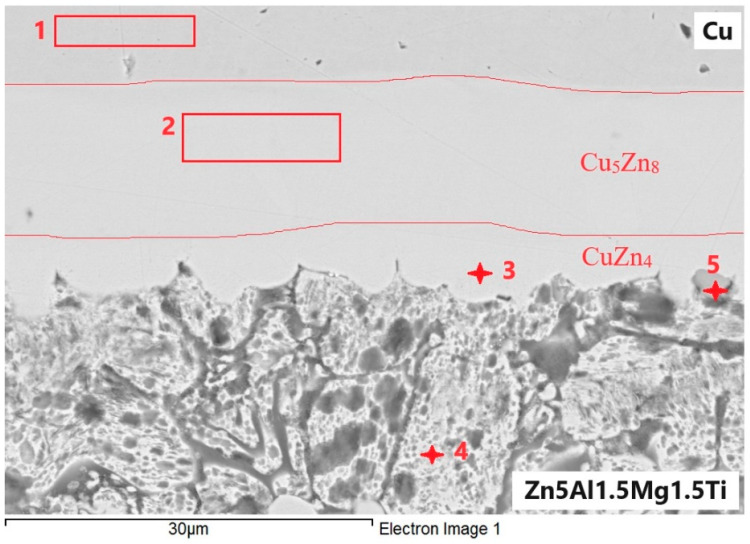
EDX point analysis of Zn5Al1.5Mg1.5Ti/Cu joint.

At the boundary of the Zn5Al1.5Mg1.5Ti/Cu joint, an undulated boundary was formed due to the application of ultrasonic power (Figure 14). The regions 1 to 5, presented in Table 6, were analyzed.

Spectrum 1 represents the zone of the Cu substrate.

Spectrum 2 corresponds to the zone that runs parallel to the Cu/solder boundary. It is unequivocally identified as the Cu_5_Zn_8_ zone, formed by the interaction of Zn and Cu, with diffusion of Al and Mg from the solder occurring in this zone.

Spectrum 3 consists of a zone that is parallel to the solder/Cu substrate boundary, where the CuZn_4_ phase is also clearly present. Additionally, slight contamination with Al and O, as well as slight contamination with Mg from the solder, was observed.

Spectrum 4 is formed of eutectics or eutectoid with a high Zn content (83 at.%), along with Cu and Al (both with 3.7 at.%). The surface is slightly oxidized.

Spectrum 5 consists of a ternary phase of type T (see diagram Al-Ti-Zn above), with the cross-section surface also being slightly oxidized.

**Table 6:** Results from the energy–dispersion analysis of Zn5Al1.5Mg1.5Ti/Cu joint.

Spectrum	O [at.%]	Mg [at.%]	Al [at.%]	Ti [at.%]	Cu [at.%]	Zn [at.%]	Solder Component
1	2.03	0	0	0	96.62	1.35	Substrate Cu
2	2.45	0.69	7.72	0	37.78	51.36	Cu_5_Zn_8_
3	1.87	0.44	2.25	0	17.8	77.64	CuZn_4_
4	8.8	0.4	3.79	0	3.68	83.33	Eutectoid
5	3.98	0	24.88	21.27	0	49.87	T-type ternary phase

**Figure 15:**
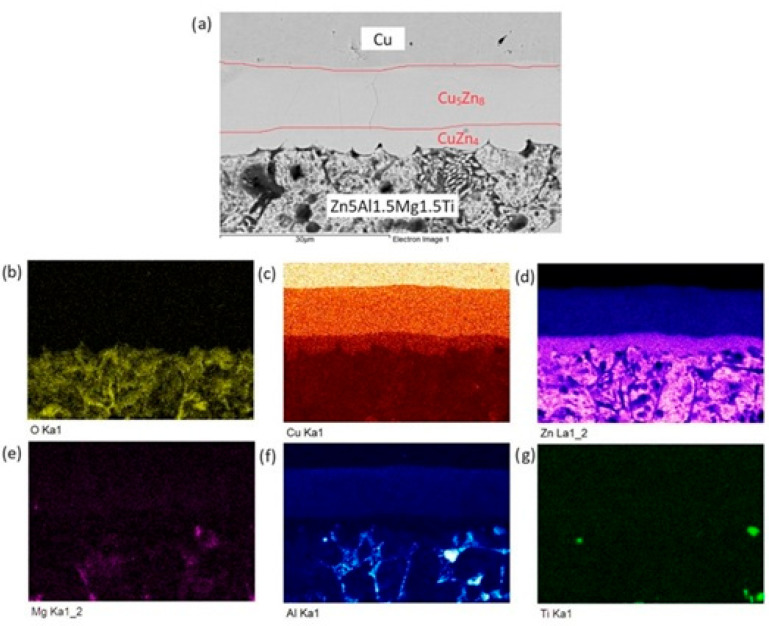
Planar distribution of elements in the boundaries of Zn5Al1.5Mg1.5Ti/Cu joints: (**a**) joint microstructure; (**b**) O; (**c**) Cu; (**d**) Zn; (**e**) Mg; (**f**) Al and (**g**) Ti.

The distribution of individual elements across the boundary zones of the Zn5Al1.5Mg1.5Ti/Cu joint is clearly visible in the qualitative summary map of the chemical analysis and is formed mainly of a uniformly distributed solder matrix (Zn). From Figure 15, we can observe the formation of the Cu_5_Zn_8_ and CuZn_4_ phases depicted in color.

**Figure 16:**
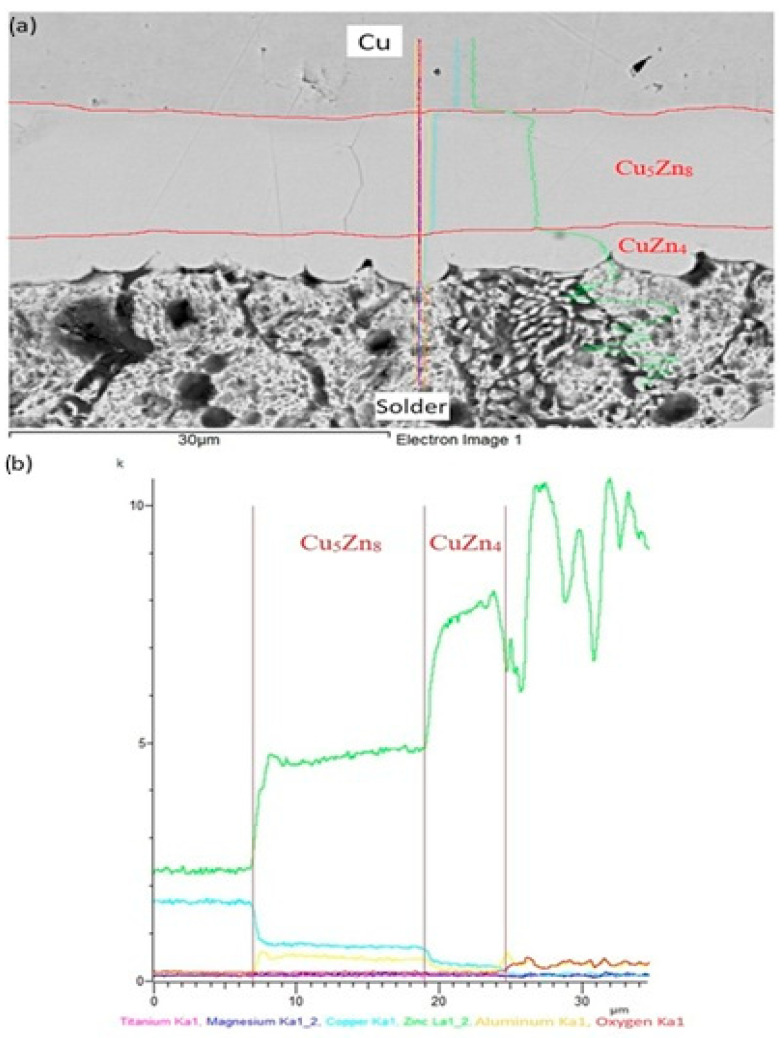
Line EDX analysis of soldered joint of Zn5Al1.5Mg1.5Ti/Cu: (**a**) joint microstructure; (**b**) concentration profiles of individual elements across the boundary.

The line analyses shown in Figure 16 represent the concentration profiles across the boundary of the Zn5Al1.5Mg1.5Ti/Cu joint, over its entire length of 30 μm. The interaction of molten alloy with the Cu substrate has resulted in the partial dissolution of copper in the solder due to diffusion, leading to the formation of a new phase (Cu_5_Zn_8_) approximately 12 µm wide, and is circumscribed by the concentration profiles. Behind this, the identified CuZn_4_ phase appears, measuring approximately 5 µm wide.

### 3.6. Shear Strength of the Soldered Joints

**Figure 17:**
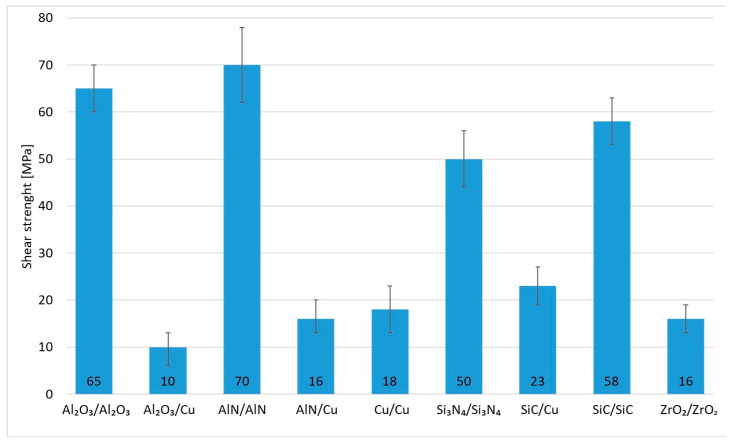
Shear strength of soldered joints fabricated by use of Zn5Al1.5Mg1.5Ti solder.

The research in this work focused on soldering Al_2_O_3_ ceramics and Cu substrates. The aim of this study was to assess the suitability of using an active solder of the type Zn5Al1.5Mg1.5Ti. Measurements were performed on three samples. In the case of the studied combination of Al_2_O_3_ ceramic and Cu substrate using Zn5Al1.5Mg1.5Ti solder, an average strength of 10 MPa (Figure 17) was attained. Given the versatility of Zn5Al1.5Mg1.5Ti solder and its potential applications in technical practice, the shear testing was also extended to other ceramic materials (Al_2_O_3_, AlN, Si_3_N_4_, SiC and ZrO_2_) in addition to Cu.

**Figure 18:**
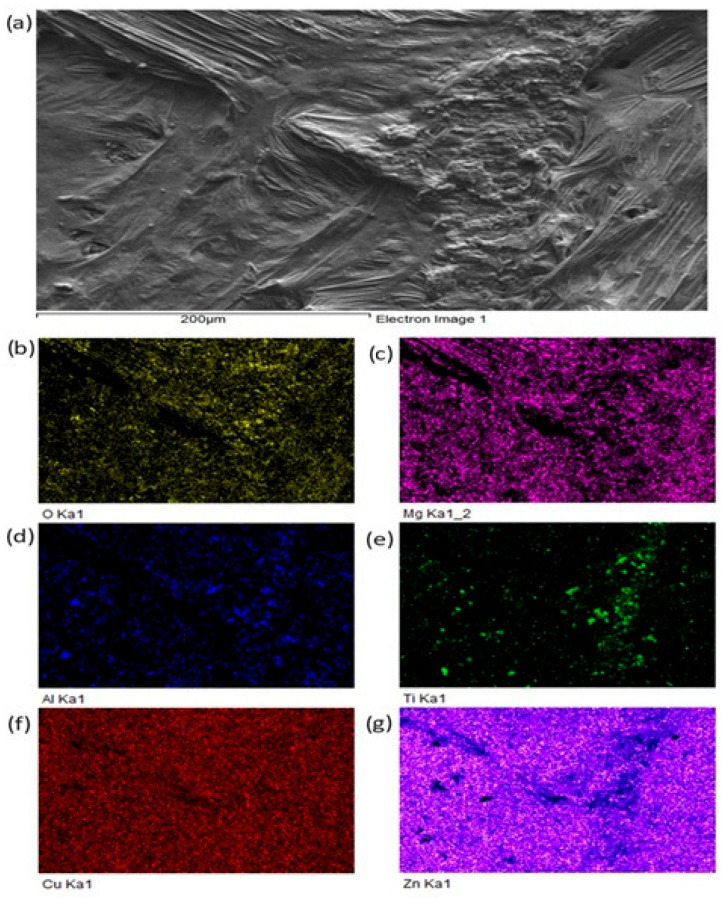
Fractured surface of Al_2_O_3_/Zn5Al1.5Mg1.5Ti joint and the planar distribution of individual elements: (**a**) fracture morphology, (**b**) O, (**c**) Mg, (**d**) Al, (**e**) Ti, (**f**) Cu, (**g**) Zn.

For a more precise identification of the mechanism of bond formation, the fractured surface of the joint was analyzed. Figure 18 shows the documented fractured surface from the boundary of the Al_2_O_3_/Zn5Al1.5Mg1.5Ti/Cu joint. It is obvious that the fractured surface from the side of the Al_2_O_3_ ceramics remained almost fully covered with solder. Besides that, small grains of Al_2_O_3_ can also be observed locally on the fractured surface. The fracture occurred in the solder. An analysis of planar distribution of Al, O, Cu, Mg, Zn and Ti elements on the fractured surface was also performed, and it is documented in Figure 18b–g. The planar distribution of magnesium, shown in Figure 18c, suggests that magnesium was present in the entire volume of solder/ceramics. Similarly, the distribution of titanium, shown in Figure 18e, suggests that titanium is locally surrounding the Al grains (Figure 18c), which represent the Al_2_O_3_ ceramics. This analysis has proved that Mg and Ti significantly contribute to bond formation. Zinc (Figure 18g) is uniformly distributed over the entire fracture.

**Figure 19:**
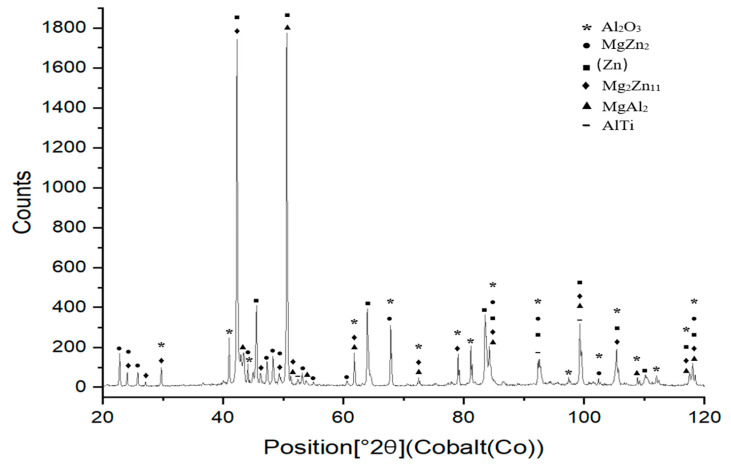
XRD analysis of fractured surface of Al_2_O_3_/Zn5Al1.5Mg1.5Ti/Cu joint.

XRD analysis was performed to examine the phases formed in the boundary of the Al_2_O_3_/Zn5Al1.5Mg1.5Ti/Cu joint (Figure 19). The presence of the Al_2_O_3_ ceramic and Cu substrate on the fractured surface was confirmed. Additionally, solid solutions of zinc and magnesium phases, such as Mg_2_Zn_11_ and MgZn_2_, were observed. In addition, AlTi and MgAl_2_ phases were identified on the fractured surface, resulting from the interaction of molten solder with the surface of the Al_2_O_3_ ceramic substrate.

## 4. Conclusions

The aim of this study was to assess the active solder type Zn5Al1.5Mg1.5Ti, intended for soldering the combination of Al_2_O_3_ ceramics and Cu substrate. To improve the wettability of the ceramic material by the Zn-Al-Mg-Ti solder, soldering with ultrasonic soldering was employed. The achieved results can be characterized as follows:

- TG/DTA analysis was applied to determine the melting point of the solder, which was confirmed. This proved the occurrence of two transformations: namely, the eutectic and eutectoid reactions. The liquidus temperature was determined to be 380 °C.
- The microstructure of the Zn-Al-Mg-Ti solder consisted of solid solution (Al), solid solution (Zn) and the MgZn_2_ phase, as evidenced by XRD analysis of the solder. The EDX analysis also revealed the presence of TiAl_3_ and Ti_3_Al phases. The ICP-AES analysis indicated the following average composition of the alloy: 95 wt. % Zn; 3.01 wt. % Al; 0.56 wt. % Ti and 1.43 wt. % Mg.
- The tensile strength of Zn-Al-Mg-Ti soldering alloy varied from 71 to 108 MPa.
- The bond between the ceramics and the Zn5Al1.5Mg1.5Ti solder was formed as follows: owing to ultrasound activation, the particles of zinc, magnesium and titanium were distributed at the boundary with the ceramic substrate, resulting in increased surface roughening and wettability. The line EDX analysis confirmed a high magnesium content at the boundary with the Al_2_O_3_ surface. Mg layer thickness in the boundary was approximately 2 μm.
- The joint boundary between the Zn5Al1.5Mg1.5Ti solder and the Cu substrate was formed mainly of Zn and Cu elements. Magnesium at the boundary did not contribute to bond formation with the Cu substrate. The transition zone between the solder and the substrate was composed of the Cu_5_Zn_8_ and CuZn_4_ phases.
- The average shear strength of the Al_2_O_3_/Cu joint fabricated using Zn5Al1.5Mg1.5Ti solder was 10 MPa.
- The research on soldering the combination of Al_2_O_3_ ceramics with the Cu substrate using Zn5Al1.5Mg1.5Ti solder has proved the suitability of the selected soldering alloy for practical applications.

Future research should focus on optimizing the Ti content to achieve a balance between mechanical strength and interfacial bonding quality. Additional studies could explore the effect of alternative alloying elements, different processing conditions or post-soldering heat treatments to further enhance the performance of Zn-based solders.

Furthermore, the results of this study can be applied to the development of lead-free soldering materials for high-temperature applications, particularly in power electronics, automotive electronics and renewable energy systems, where solder joints must withstand harsh operating conditions. Understanding the trade-off between mechanical strength and interface adhesion is crucial for designing next-generation solder alloys.

## Figures and Tables

**Table 1:** Composition of Zn-Al-Mg-Ti alloy and the results of chemical analysis performed by ICP-AES method [wt. %].

Sample	Charge [wt. %]				ICP-AES [wt. %]			
	Zn	Al	Mg	Ti	Zn	Al	Mg	Ti
Zn5Al1.5Mg1.5Ti	92.0	5	1.5	1.5	95	3.01	1.43	0.56

**Table 2:** Ultrasound parameters.

Ultrasound power	[W]	400
Working frequency	[kHz]	40
Amplitude	[μm]	2
Soldering temperature	[°C]	380
Ultrasound exposure time	[s]	5

## Data Availability

The original contributions presented in this study are included in the article. Further inquiries can be directed to the corresponding author.
